# Systemic changes in cell size throughout the body of *Drosophila melanogaster* associated with mutations in molecular cell cycle regulators

**DOI:** 10.1038/s41598-023-34674-y

**Published:** 2023-05-09

**Authors:** Valeriya Privalova, Anna Maria Labecka, Ewa Szlachcic, Anna Sikorska, Marcin Czarnoleski

**Affiliations:** Life History Evolution Group, Institute of Environmental Sciences, Faculty of Biology, Jagiellonian University, Gronostajowa 7, 30-387 Kraków, Poland

**Keywords:** TOR signalling, Evolutionary developmental biology, Ecophysiology, Evolutionary biology, Animal physiology, Cell biology, Evolution, Zoology

## Abstract

Along with different life strategies, organisms have evolved dramatic cellular composition differences. Understanding the molecular basis and fitness effects of these differences is key to elucidating the fundamental characteristics of life. TOR/insulin pathways are key regulators of cell size, but whether their activity determines cell size in a systemic or tissue-specific manner awaits exploration. To that end, we measured cells in four tissues in genetically modified *Drosophila melanogaster* (*rictor*^*Δ2*^ and *Mnt*^*1*^) and corresponding controls. While *rictor*^*Δ2*^ flies lacked the Rictor protein in TOR complex 2, downregulating the functions of this element in TOR/insulin pathways, *Mnt*^*1*^ flies lacked the transcriptional regulator protein Mnt, weakening the suppression of downstream signalling from TOR/insulin pathways. *rictor*^*Δ2*^ flies had smaller epidermal (leg and wing) and ommatidial cells and *Mnt*^*1*^ flies had larger cells in these tissues than the controls. Females had consistently larger cells than males in the three tissue types. In contrast, dorsal longitudinal flight muscle cells (measured only in males) were not altered by mutations. We suggest that mutations in cell cycle control pathways drive the evolution of systemic changes in cell size throughout the body, but additional mechanisms shape the cellular composition of some tissues independent of these mutations.

## Introduction

Cellularity is one of the most distinctive features of life; even cell-free life forms such as viruses and viroids complete their life cycles by “taking over” the metabolic machinery of host cells. The increased diversification of the life strategies, body plans and sizes of life forms on Earth was accompanied by dramatic changes in the cellular structure of organisms’ bodies. While bacteria and protists are organisms with single “universal” cells, giant organisms such as sequoias and blue whales have billions of highly specialised cells of diverse types. Within metazoans, the emergence of more complex forms was closely linked to an increase in the diversity of cell types with distinct phenotypes and specialised functions^1,2^. For example, the body of a 70-kg human consists of approximately 37 trillion cells^3,4^ of at least 411 different types^5^. The most fundamental properties of cells, such as their size, shape and organelle content, directly reflect cell-specific functions in the body^6,7^ and therefore remain under the control of natural selection^5^. Thus, cells are subject to certain constraints and fit together like a jigsaw puzzle to form tissues and organs, resulting in cells of a given type having roughly defined sizes and shapes^8,9^. Nevertheless, cell sizes in specific tissue types do not remain constant when species or populations undergo evolutionary diversification^10,11^ or when genotypes develop in different environments^11,12^. Accordingly, we do not fully understand everything that determines the cellular composition of organisms, and we are only starting to elucidate the fitness consequences of this fundamental property of life^13–15^.

The development of a metazoan body entails interplay between myriad external and internal cues and the massive exchange of chemical information among proliferating, growing and differentiating cells^16,17^. Although we are just beginning to understand this complex crosstalk, advances in technology have allowed us to identify a number of molecular signalling pathways involved in controlling the cellular composition of tissues. These pathways rely on receptors that sense information about organismal states (e.g., hormones, nutrients, metabolites) and the activity of various protein kinases, such as AMP-activated kinase (AMPK), target of rapamycin (TOR), and Hippo (Hpo) kinase, and ultimately involve an array of transcriptional regulators, such as the tandem antagonistic proteins Myc and Mnt^18–22^. In animals, two multiprotein complexes including TOR (TOR complexes 1 and 2) act as the master signalling hubs of TOR/insulin pathways, which integrate signals from upstream pathways to inform downstream pathways, ultimately regulating transcription and translation processes^23,24^. The TOR/insulin pathways are involved in monitoring the physiological states of cells and responding to oxygen and nutrient fluxes and different stress sources, such as heat, osmolarity and DNA damage^25–29^. Nutrient-mediated changes in TOR activity have been shown to affect development in *Drosophila melanogaster*^30^, *Caenorhabditis elegans*^31^, and other organisms^32^. It has also been shown that dietary restriction resulting in the downregulation of TOR activity promotes autophagy and can extend lifespan^33–35^. Therefore, the TOR/insulin pathways are crucial for maintaining cellular and organismal homeostasis, and defects in these pathways can lead to unhealthy conditions and diseases such as obesity, diabetes and cancer^36,37^.

The evolution of TOR/insulin pathways, life history strategies, and the cellular composition of organisms appear to be interrelated. On the macroevolutionary time scale, evidence suggests that eukaryotic cells occurred in conjunction with the emergence of the TOR pathway, and with the appearance of animals, the TOR pathway began to be additionally regulated by the insulin pathway^20,38^. At the microevolutionary level, the continental expansion of *D. melanogaster* flies resulted in the evolution of latitudinal clines in cell and body sizes associated with shifts in the activity of the TOR/insulin pathways^39,40^. The cellular composition of the metazoan body reflects the collective effects of cell cycle control pathways in individual cells. However, it is not clear whether these effects result in systemic changes in cell size throughout the body, with different tissues following the same pattern, or whether they depend mainly on local processes aimed at matching cellular properties to tissue-specific functions, with each tissue following its own pattern. To investigate whether the modulation of TOR/insulin pathways leads to either correlated or independent changes in cell size in different tissues, we studied cell size in four genetic lines of *D. melanogaster* engineered via a method involving the imprecise excision of a P-element: the *rictor*^*Δ2*^ mutant line and corresponding control (EY08986, see^41^ for methodological details of mutant creation) and the *Mnt*^*1*^ mutant line and corresponding control (EP(X)1559, see^42^ for methodological details of mutant creation). The Rictor protein is a structural component of TOR complex 2, but not TOR complex 1^23,28^. Therefore, we hypothesized that Rictor protein deficiency in the *rictor*^*Δ2*^ mutant reduces the functioning of TOR complex 2 (e.g., insulin sensing, actin organization) but does not directly affect the functioning of TOR complex 1 (e.g., amino acid sensing, protein synthesis). However, the mutation may have more complex effects given that the activity of the two TOR complexes can be interlinked by crosstalk signalling^43,44^. The transcriptional regulator protein Mnt is mainly involved in translation control by attenuating signals from activated TOR/insulin pathways^45^. Therefore, we hypothesized that Mnt protein deficiency in the *Mnt*^*1*^ mutant does not directly alter the function of TOR complexes 1 and 2 but leads to weak suppression of downstream signalling originating from TOR/insulin pathways, thereby upregulating protein synthesis. After raising our flies under common garden conditions, we performed cell size measurements in four different organs: epidermal cells in legs and wings, ommatidial cells in eyes, and cells of dorsal longitudinal flight muscles in the thorax (a subset of indirect flight muscles; hereafter flight muscles). The studied cell types originate from two germ layers, ectoderm (epithelia in legs and wings and cells forming ommatidia in the compound eyes) and mesoderm (flight muscles)^46^. This allowed us to gather exceptionally rich information on cell size in the *rictor*^*Δ2*^ and *Mnt*^*1*^ flies, which represent two cases of a wide range of genetic modifications in *D. melanogaster* that were primarily engineered for research on tumorigenesis and cancer therapies^41,42,47^. Choosing these two mutations for our study, we considered that they result in viable flies in homozygous states, which provided us with a convenient system for large-scale cell size measurements. Importantly, among their various effects, the selected mutations were expected to impose cell size shifts in two opposite directions, with *rictor*^*Δ2*^ serving as a model of small-celled flies and *Mnt*^*1*^ as a model of large-celled flies. To date, *rictor*^*Δ2*^ flies (also known as *rictor D2*, *rictor*^*2*^, and *rictor*^*Δ2*^) have been reported to show 10% reductions in body mass and wing size^41^, decreases in total neuron length and arbour size, and a lower final number of neurons^48^ than wild-type flies. *Mnt*^*1*^ (also known as *dmnt*^*1*^ and *mnt*^*1*^) flies were reported to exhibit a 20% greater body mass, 18% fewer epidermal cells per wing area unit^42^, and larger ommatidia^49^ than wild-type flies. Previous research on the phenotypic consequences of these mutations has not focused on multiple cell types simultaneously, leaving it unclear whether cell size changes imposed by each mutation occur throughout the body or locally in certain tissue types. Crucially, while cell size variance between organisms is gaining scientific attention^50–52^, to date, very few studies have considered different cell types simultaneously, especially at the intraspecies level (but see^53–57^). As a result, it remains unknown whether the individual differences in cell size revealed by studies of single cell types are indicative of general phenomena in the organization of whole organisms or only of local tendencies in single tissues.

## Materials and methods

### Animals

The studied lines of *D. melanogaster* were obtained courtesy of Prof. Ville Hietekangas (University of Helsinki, Finland), who provided *rictor*^*Δ2*^ and its control; Prof. Peter Gallant (University of Wurzburg, Germany), who provided *Mnt*^*1*^; and Prof. Robert N. Eisenman (Fred Hutchinson Cancer Research Center, the United States of America) who provided the control for *Mnt*^*1*^. The flies were maintained in a fly stock of the Institute of Environmental Sciences, Jagiellonian University, Kraków, Poland. The stock was kept under controlled conditions in thermal cabinets (Pol-Eko-Aparatura, Wodzisław Śląski, Poland) set to 20.5 °C and a 12 h:12 h L:D photoperiod. Relative humidity inside the cabinets was sustained at a stable 70% with containers of water placed in the cabinets. The stock flies were kept in 68-mL fly vials with polyurethane foam plugs containing 20 mL of standard cornmeal yeast food (Bloomington Drosophila Stock Center, Bloomington, IN, USA), and the same conditions were applied to all steps of our study here. Every three weeks, stock flies were placed for five days in fresh vials for egg laying, which maintained nonoverlapping generations. For the purpose of our study, we used stock flies to generate two consecutive generations under controlled larval density, boosting the number of vials with flies and thus securing an adequate number of flies for cell size measurements. Upon each of these two transfers, we created multiple mating groups of 10 females with 4 males per genetic line (two mutants with their respective controls) and placed each group for 48 h in a fresh vial for egg laying.

### Cell size

**Figure 1:**
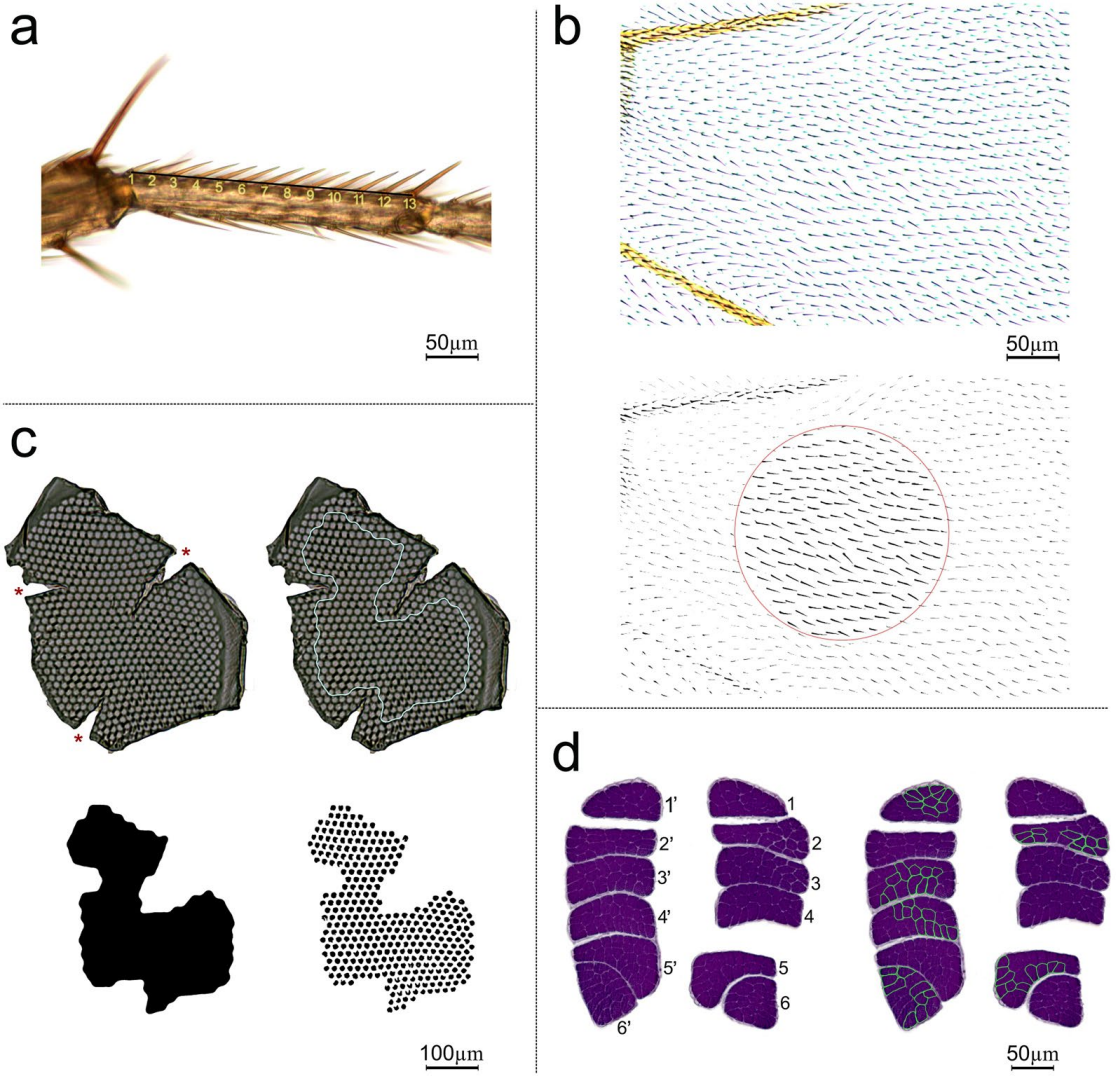
Procedures for the determination of proxies of cell size in four cell types of *Drosophila melanogaster*. (**a**) The size of epidermal cells in the leg was estimated based on the bristle number per length unit of the basitarsus (first tarsal segment) of the left second leg. The numbers represent the counts of the bristles of the eighth longitudinal row. The black line represents the distance between the middle of the root of the first and last bristles. (**b**) The size of epidermal cells in the wing was estimated based on the trichome number of the dorsal wing blade of the left wing in a defined circular area of 0.031 mm^2^ (red) located between the cubital and distal veins. (**c**) The size of ommatidial cells in the eye was estimated based on the mean ommatidial area determined from UV-glue replicas of eyes flattened on microscopic slides. The red asterisks indicate the incisions in the replicas. The light blue line indicates the area selected for measurement. Estimation of the total area with ommatidia; the number of ommatidia within the area are depicted with black patterns. (**d**) The cell size of indirect flight muscles was estimated based on the mean cross-sectional area of fibres in dorsal longitudinal muscles in the thorax. The numbers represent six muscle bundles of two rows on the right (1–6) and left (1′–6′) sides of the thorax. Cross-sections of individual fibres with well-defined borders (outlined in light green) were used for measurements.

Histological analyses were performed 2–10 days after eclosion on flies originating from the second generation, which was raised with controlled larval density, following our previously published histological methods for flies^58^. The flies were sampled evenly from all available vials, which eliminated any possibility of bias in the results that could be caused by potential random effects of vials. A collected fly was briefly anaesthetized with CO_2_ with the help of a flypad and blowgun (Genesee Scientific, San Diego, USA). We used an ocular scale in a stereomicroscope (Olympus SZX12, Olympus, Tokyo, Japan) to measure the distance (mm) from a thoracic neck edge to the tip of the scutellum, our measure of thorax length and a proxy of body size. Then, we performed body dissections with a microtome knife and forceps to acquire different body parts with four tissues (Fig. 1): the left middle leg with epidermal cells, the left wing with epidermal cells, the head with cells that form ommatidia, and the thorax with dorsal longitudinal indirect flight muscles. For technical reasons, the legs, wings, and heads were obtained from the same individual flies (80 samples of legs, wings, and eyes: 10 females and 10 males per genetic line), and thoraxes were obtained from other individuals (80 samples: 20 males per genetic line). For the cell size measurements, the organ samples were imaged under a light microscope in a bright field (Eclipse 80i, Nikon, Tokyo, Japan) with a camera (Axio Cam MRc5, Zeiss, Oberkochen, Germany) and ZEN software (ver. 2011, Zeiss).

The fly legs were stored individually in Eppendorf tubes containing 1 ml of 70% ethanol (Linegal Chemicals, Warszawa, Poland). For measurements (Fig. 1a), each leg was placed on a glass microscopy slide, and an image of its basitarsus was taken with a 20 × objective under the microscope. Using ZEN software (Zeiss), the distance (µm) between the first and last bristles of the 8th row of the basitarsus, according to the nomenclature of Hannah-Alava^59^, was measured, and the total number of bristles was counted. Held^60^ demonstrated that in *Drosophila,* the number of bristles in a row varies co-ordinately with the number of cells along the basitarsus, while the bristle interval is coordinated with cell diameter. Thus, to obtain a proxy of mean epidermal cell size in the leg (µm), we divided the distance between the first and the last bristles by the total number of bristles in the row. For consistency across all cell types, the linear measures of leg cells were squared (μm^2^).

The wings were freeze-stored individually in Eppendorf tubes at − 20 °C. For the measurements (Fig. 1b), a wing was placed on a microscopic glass slide in a drop of ST Ultra (Leica, Wetzlar, Germany), flattened, and then covered with a coverslip mounted with CV Ultra (Leica). The wings were imaged under 20 × objective magnification. Following previously published methods^61,62^, we determined trichome density in a defined area of a wing blade, as each trichome represents one epidermal cell^63^. The analysis was performed on binarized and segmented images of the dorsal wing blade with a help of standard tools (Channels, Threshold, Analyze Particles) in ImageJ software (National Institutes of Health (NIH), Bethesda, MD, USA), automated with a macro that applied each tool to multiple images. In the first step, an image of the wing blade was split to three RGB channels, and the image from the green channel was binarized and used in subsequent analyses. A defined circular area of 0.031 mm^2^ was burned into the image between the cubital and distal veins of the wing blade. We manually wiped out trichomes that were rooted outside the circle and separated interconnected trichomes within the circle. Then, using the ‘Analyze particles tool’, we obtained the number of particles (trichomes) within the circle. The area of the circle was then divided by the number of counted trichomes to calculate the mean size of epidermal cells in the wing (µm^2^).

After the dissections, the heads were preserved individually in Eppendorf tubes containing 1 ml of 20% methanol (POCH, Gliwice, Poland). Since *Drosophila* eyes are composed of ommatidia, which are formed by a fixed number of cells^64^, we used the area of the ommatidium facet as a proxy of cell size following the methods of earlier works^27,65,66^. To measure the size of ommatidia (Fig. 1c), we followed our previously published methods for beetles^66^, modified for flies: the head was impaled on a thin entomological pin, and both eyes were covered with a thin layer of transparent 5SecondFix UV glue. After drying with a UV lamp (Ontel Products, Fairfield, USA), the eye replica was removed with an entomological pin and forceps, incised at three points of the outer edge, and flattened on a microscopic slide in a drop of glycerine (POCH). To facilitate flattening, the replica was covered with a coverslip loaded with a fishing weight (100 g) for 24 h. Thereafter, the edges of the coverslip were sealed with nail polish. Images of the eye replicas were taken under 10 × objective magnification. The average ommatidium size (µm^2^) was calculated with a help of the automatized methods of Scharmm et al.^66^ integrated into ImageJ (NIH).

The thoraxes were fixed in Duboscq-Brasil’s solution for 2 h and placed in a beaker with water on a hot plate for 10 min, according to the modified methodology of Barbosa, Berry and Kary^67^. Following fixation, a sample was washed in PBSTX (Carl Roth, Karlsruhe, Germany); dehydrated in a graded ethanol series (Linegal Chemicals), butanol (Chempur, Piekary Śląskie, Poland), and isopropanol (Leica); cleared in pure Clearene (Leica) and then in a mixture of Clearene (Leica) and Paraplast Plus (Leica); and embedded in Paraplast Plus (Leica). Serial cross-sections (6 μm thick) of the thorax in its dorso-ventral plane were cut with a rotary microtome (Hyrax M55, Zeiss). The histological slides were deparaffinized in Clearene (Leica), rehydrated in isopropanol (Leica) and ethanol solutions with decreasing concentrations (Linegal), and stained with Ehrlich haematoxylin (Carl Roth) for two minutes and with Gömöri trichrome (BioOptica, Milano, Italy) for 20 min. After that, the slides were washed in 0.2% acetic acid (POCH), dehydrated in 96% ethanol (Linegal), cleared in a mixture of phenol (Chempur) and xylene (POCH), then in pure xylene (POCH), and embedded in CV Mount (Leica). The cross-sections of the flight muscles were imaged under 20 × objective magnification (Fig. 1d). For each fly, we imaged six muscle bundles among the bundles visible in two rows on the right and left sides of the thorax: 1–6 and 1’–6’. For the measurements, we chose one bundle from each pair. Afterwards, we used ImageJ (NIH) to outline the cross-sections of the individual cells (fibres) to measure the cross-sectional area of muscle cells (μm^2^). Note that in the case of poor visibility of borders between two adjusted cells, we outlined two cells together and then divided the total area by two.

After all the measurements, each fly was characterized by the mean cell size value for each cell type. Overall, the calculation of mean cell sizes involved the following number of cell-size related structures (per individual fly): legs—from 11 to 17 bristles; wings—from 154 to 243 trichomes; eyes—from 69 to 673 ommatidia; and thorax—60 muscle cells. Note that our final analyses of flight muscle cells involved 42 out of 80 thorax samples, as we discarded nonperpendicular muscle cross-sections (see Supplementary Tables 1S and 2S online for detailed descriptive information of our data).

### Statistical analysis

Statistical analysis was conducted in R 4.1.3 software^68^ using the stats package^68^ for ANOVA and the emmeans^69^ and ggplot2^70^ packages for graphics. To explore whether cell size undergoes correlated changes in different cell types, we performed ANOVA on our cell size measures in four organs, separately for each organ. Each ANOVA considered the genetic line (*rictor*^*Δ2*^ mutant, *rictor*^*Δ2*^ control, *Mnt*^*1*^ mutant, *Mnt*^*1*^ control) as a fixed grouping factor, and in the case of the analysis of the epidermal cells in legs, wings, and ommatidia, it also considered sex as another fixed factor (muscle cells were measured only in males). The ANOVAs were run with and without thorax length (our proxy of body size) as a continuous covariate. To compare specific genotypes, each ANOVA was followed by contrast analysis that tested a difference between each mutant (*rictor*^*Δ2*^ and *Mnt*^*1*^) and its respective control. We checked normality with the Kolmogorov‒Smirnov test and the homogeneity of variance with Levene’s test. Our data on flight muscles deviated slightly from this assumption, so we additionally performed a nonparametric Kruskal‒Wallis test, followed by pairwise comparisons between mutant and control flies with the Wilcoxon test, using the stats package^68^. Hereafter, we report the ANOVA results for consistency in all tissues because the nonparametric analysis (Supplementary Table 3S online) led to the same conclusion as the ANOVA.

## Results

**Figure 2:**
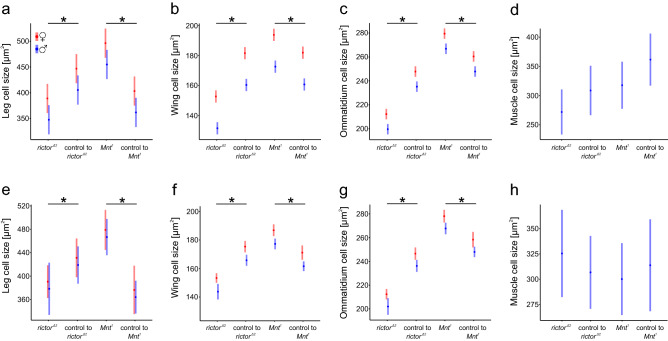
Means with 95% confidence intervals modelled by ANOVA for cell size in four tissues of *Drosophila melanogaster* (see Table 1 for statistical models). The asterisks indicate statistical significance in a contrast analysis comparing each mutant with its respective control. The analysis of cell sizes in legs, wings and eyes yielded similar results: *rictor*^*Δ2*^ flies had smaller epidermal cells in (**a**) legs and (**b**) wings and smaller (**c**) ommatidial cells, while *Mnt*^*1*^ flies had larger epidermal cells in (**a**) legs and (**b**) wings and larger (**c**) ommatidial cells in comparison to their respective controls. Females appeared to have larger cells than males in these organs (**a**–**c**). The cell size of flight muscles (measured only in males) did not differ between *rictor*^*Δ2*^ and the corresponding control or between *Mnt*^*1*^ and the corresponding control (**d**). Controlling for thorax length as a covariate (**e**–**g**) yielded similar results: the *rictor*^*Δ2*^ group showed decreased cell sizes in (**e**) legs, (**f**) wings and (**g**) eyes, while the *Mnt*^*1*^ group showed increased cell sizes in (**e**) legs, (**f**) wings and (**g**) eyes in comparison to their respective control groups. The effect of sex remained significant only for (**g**) ommatidial and not for epidermal tissues in (**e**) legs or (**f**) wings.

The ANOVA results for the legs, wings, and eyes (Table 1a; Fig. 2a–c) showed consistent patterns in cell size differences between the genetic lines. When compared to their respective controls, the *rictor*^*Δ2*^ mutant flies had smaller epidermal cells in legs (*p* = 0.002) and wings (*p* < 0.001) and smaller ommatidial cells in the eyes (*p* < 0.001), while *Mnt*^*1*^ mutant flies had larger epidermal cells in legs (*p* < 0.001) and wings (*p* < 0.001) and larger ommatidial cells in the eyes (*p* < 0.001). In all genotype groups, females had consistently larger cells than males in the legs (*p* = 0.002), wings (*p* < 0.001), and eyes (*p* < 0.001). The ANOVA for males’ flight muscles (Table 1a; Fig. 2d) showed significant differences in the size of this cell type among our four genetic groups. However, when we used contrast analysis comparing each mutant to its control, we did not find significant differences at the *p* = 0.05 level (*p* = 0.155 for *rictor*^*Δ2*^ vs. control and *p* = 0.199 for *Mnt*^*1*^ vs. control). Altogether, our data indicate correlated changes in cell size in three cell types driven by mutations and sex effects, with some autonomy in the regulation of cell size in one cell type.

**Table 1:** Results of ANOVA models for the cell sizes of legs, wings, eyes and flight muscles in four genetic groups of *Drosophila melanogaster* (*rictor*^*Δ2*^ mutant and its control, *Mnt*^*1*^ mutant and its control) without (a) and with the thorax length treated as a covariate (b).

Factor	*df*	Epidermal cells in the legs		Epidermal cells in the wings		Ommatidial cells in the eyes		*df*	Flight muscle cells in the thorax	
		F	*p*	F	*p*	F	*p*		F	*p*
(a)										
Genetic group	3	14.48	< 0.001	91.81	< 0.001	208.57	< 0.001	3	3.21	0.034
*rictor*^*Δ2*^ versus control	1	10.35	0.002	125.35	< 0.001	164.13	< 0.001	1	2.10	0.155
*Mnt*^*1*^ versus control	1	26.80	< 0.001	21.18	< 0.001	47.05	< 0.001	1	1.71	0.199
Sex	1	10.63	0.002	135.19	< 0.001	41.32	< 0.001	–	–	–
Residuals	75							38		
(b)										
Genetic group	3	13.51	< 0.001	65.57	< 0.001	138.72	< 0.001	3	0.32	0.811
*rictor*^*Δ2*^ versus control	1	5.68	0.020	63.40	< 0.001	58.35	< 0.001	1	0.40	0.531
*Mnt*^*1*^ versus control	1	33.64	< 0.001	70.49	< 0.001	93.27	< 0.001	1	0.40	0.531
Sex	1	0.26	0.614	0.31	0.580	29.45	< 0.001	–	–	–
Thorax length	1	17.87	< 0.001	421.7	< 0.001	219.88	< 0.001	1	27.44	< 0.001
Residuals	74							37		

The analysis included two contrasts, in which each mutant type was compared with its control. Data for legs, wings and eyes were collected for both sexes, while data for flight muscles were collected only for males.

Adding thorax length as a predictor to our ANOVA models (Table 1b, Fig. 2e–h) gave the same picture of the effects of mutations on cell size as when based on models without this variable. The *rictor*^*Δ2*^ flies showed decreased cell size in the legs (*p* = 0.020), wings (*p* < 0.001), and eyes (*p* < 0.001), while *Mnt*^*1*^ flies showed increased cell size in the legs (*p* < 0.001), wings (*p* < 0.001), and eyes (*p* < 0.001) in comparison to their respective controls. The mutations did not affect cell size in flight muscles (*p* = 0.531 for *rictor*^*Δ2*^ vs. control and *p* = 0.531 for *Mnt*^*1*^ vs. control). The analysis of these models also indicated that some of the variance in cell size was linked to individual differences in body size. This is indicated by a significant positive relationship between thorax length and the size of all cell types: the legs (*p* < 0.001), the wings (*p* < 0.001), the eyes (*p* < 0.001), and the flight muscles (*p* < 0.001) (Table 1b). Adding the thorax length to ANOVAs also weakened the significance of sex effects in models, indicating that some sex differences in cell size were associated with sexual body size dimorphism. The effect of sex, with larger cells in females than males, remained significant in the analysis of ommatidial cells in the eyes (*p* < 0.001) but not in the analysis of epidermal cells in the legs (*p* = 0.614) and the wings (*p* = 0.580) (Table 1b).

## Discussion

We are just beginning to elucidate the relative roles of systemic and cell-autonomous mechanisms that control the cellular composition of metazoans^21^ and the functional significance of even minor differences in cell phenotypes^71^. These advancements are crucial for understanding how and why cell number, cell geometry and cell size are regulated in tissues and organs and how these cellular organization characteristics of an organism shape its performance in environmental gradients. Expanding on this hot research topic, our results for *D. melanogaster* indicate that cells can undergo highly correlated changes in different tissue types while their size also remains under local tissue-specific control. In our study, the organism-wide orchestration of cell sizes manifested in two ways: developmental responses to mutations in TOR/insulin pathways and developmental processes involved in determining sex-specific phenotypes of eclosing flies. Specifically, depending on the studied organ—legs, wings or ommatidia—and the sex of the fly, a mutation in the *rictor* gene led to a 11–19% reduction in cell size, while a mutation in the *Mnt* gene resulted in a 5–29% increase in cell size. Moreover, our results showed that the organs of females were composed of cells that were 3–17% larger than those of males. At the same time, despite the correlation of cell size in the legs, wings and ommatidia, mutations in the *rictor* and *Mnt* genes did not alter cell size in the flight muscles, indicating some autonomy in building the cellular composition of this tissue, even in the presence of mutations in key elements of cell cycle control pathways. Previous studies have rarely explored cell size variation between individuals originating from the same species, especially in the context of organism-wide patterns in the cellular composition of organs and tissues, and most information about this phenomenon comes from comparisons across species^14,50–52,72^. As a contribution to filling this information gap, our data for *D. melanogaster* flies strongly suggest that the orchestration of the cellular compositions of different tissues can be a common phenomenon, even at the intraspecies level. Given our results presented here and published evidence^53–55,73^, it appears that the correlated changes in cell size throughout the body participate in the origination of sex differences and are involved in phenotypic plasticity as well as evolutionary differentiation.

As data on cell size in different tissues of the same individuals are not commonly available, it is rather challenging to find relevant information on the involvement of specific regulatory pathways in systemic changes in cell size throughout the body. For instance, although alterations in TOR/insulin pathways are known to affect cell size^21,22,25–27^, these effects have been almost exclusively studied in single cell types thus far. Our recent experiments on *D. melanogaster* demonstrated that a larval diet enriched with rapamycin, a chemical compound that blocks TOR complex 1, resulted in adult *D. melanogaster* with consistently smaller cells in five different organs^58^. Consistent with these effects of environmentally driven alterations in TOR/insulin pathways, we found here that genetic alterations in two other components of these pathways, namely, the lack of one of two functional proteins, either Rictor or Mnt, led to the organism-wide correlation of cell size. Together with TOR, the Rictor protein forms TOR complex 2, which has been shown to play a role in cell survival, proliferation and actin reorganization^74^. Although we still lack a complete picture of the signalling networks that are regulated by TOR complex 2, there is evidence that it is crucially involved in cell growth^23,75^, which was also supported by our results showing that the shortage of Rictor protein retarded cell growth, which reduced cell size. TOR complex 2 phosphorylates several protein kinases within the AGC family, such as protein kinase B (PKB, also known as Akt), serum and glucocorticoid-regulated kinase (SGK), and protein kinase C (PKC)^43,76,77^. The knockdown of the *rictor* gene reduces TOR complex 2 activity^78^ and may suppress its downstream effectors, such as Akt^77,79^, which regulates a variety of cellular processes, such as growth, proliferation, survival and metabolism^44^. Signalling from TOR complex 2 is also received by the transcription factor Myc, which promotes cellular growth mainly by modulating ribosome biogenesis and protein synthesis in response to TOR activity^75,80^. Importantly, the regulation of Myc is additionally controlled by the antagonizing effects of the transcriptional repressor Mnt^42,81,82^, which was not produced in our *Mnt*^*1*^ mutant. Indeed, we observed that the shortage of this regulatory element led to increased cell growth, leading to a correlated cell size increase throughout different tissue types.

Our study provides evidence of cell size orchestration throughout the body but also indicates the importance of some tissue-specific mechanisms in the regulation of cell size. Our results show that the two sexes showed consistent differences in cell size, despite changes in cell size driven by the studied mutations. Cell-autonomous mechanisms have been recently reported to play a role in sex-dependent regulation of cell size in *D. melanogaster*. A *transformer* gene is involved in developing sex differences in cell size, and its product, expressed only in females, is associated with systemic signalling networks via TOR/insulin pathways^83–85^. In effect, sex differences in cell size disappear in *D. melanogaster* after simultaneous downregulation of the activity of both the TOR and insulin pathways but not by downregulation of TOR alone^83^. Another indication of autonomous cell size control in our data is the nonresponsiveness of muscle cells to the *rictor*^*Δ2*^ and *Mnt*^*1*^ mutations. This result contrasts with recent findings showing that the downregulation of TOR activity during the larval period induced by a rapamycin-enriched diet causes concerted cell size changes in different tissues of adult flies, including flight muscles^58^. However, a deficiency of the Rictor protein downregulates TOR complex 2, while the presence of rapamycin downregulates TOR complex 1. We can only speculate about the mechanisms that may confer some independence in the cell size control of flight muscles in the presence of mutations in TOR/insulin pathways. Active flight has evolved independently multiple times, with insects being the only flying invertebrates on Earth. Most flying insects, including Diptera, use asynchronous indirect flight muscles, which we have studied here, and are able to power high frequency wing beats owing to the effects of resonance and deformations of the thoracic exoskeleton as well as the unique way of stimulating muscle contractions by neurons^86^. Cells that form indirect flight muscles—one of the most metabolically active tissues identified among any organism^87^—are therefore specialized in superfast contraction and production of force by relatively small wings. Although the molecular mechanisms that underlie the formation of larval muscles in *Drosophila* are well defined, the mechanisms of muscle formation in adult flies are much more poorly understood^88,89^. Not surprisingly, upon pupation and during metamorphosis, most fly muscles are organized de novo following the canonical path of myogenesis^90^. In contrast, the type of flight muscles that we considered in our study, namely, dorsal longitudinal muscles, are not fully generated de novo, but they emerge in adults as transformed larval muscles. The myofibers dedifferentiate and develop new specifications, and the multinucleated cells, precisely the syncytia that are called muscle cells (fibres), are then formed by myoblast fusion, resulting in an increase in muscle cell size^90^. We consider that the process of myoblast merging can at least partially be involved in the autonomy of muscle cell size, as suggested by our results. It is possible that in this study the mutations affected the size of precursor muscle cells (myoblasts), but if the fusion simultaneously involved an increased number of smaller myoblasts or a decreased number of larger myoblasts, then the size of the entire muscle fibre could remain unaltered. Interestingly, there is also evidence suggesting that endoreplication should also be considered when discussing changes in the physiology of muscle tissue, especially given that the regulation of endoreplication involves TOR/insulin signalling and Myc protein^91,92^. Unfortunately, we have not studied this phenomenon here, but recent studies suggest that variation in nuclear size and ploidy, both altered by endoreplication, seem not to be enough to induce or dissuade the growth of muscle cells^93^. It is becoming apparent that many additional signals that serve to modulate, e.g., as in the synthesis of proteins, ribosomes, and mitochondria, take a simultaneous part in promoting or obstructing the size increase of the cells that form muscle tissues^94^, and this remains to be explored by future studies.

Environments vary across space and time, which means that organisms, together with the cells that make up their tissues, function in different physiological states. The TOR/insulin pathways are “decision makers” that trigger physiological and life history responses according to incoming information, which ultimately shapes Darwinian fitness under the given conditions. As indicated by the theory of optimal cell size (TOCS)^50,61,95–97^ (see also the review by Kozłowski et al.^13^), the adaptive responses to environmental challenges may involve cell size regulation aimed at meeting metabolic demands (ATP, structural components) through the supply of oxygen and resources. From this perspective, the cellular composition of a body results from optimizing the costs and benefits arising from different cell sizes, largely dictated by the cost of plasma membrane maintenance and the capacity of cells to perform anabolic and catabolic processes. An increase in the cell surface area due to small cell sizes should increase the expenditures required for maintaining ionic gradients on plasma membranes and membrane remodelling. In support of this hypothesis, evidence shows that mass-specific metabolic rates tend to decrease in large-celled organisms^50,51,98^. On the other hand, small cells should maintain a better transport capacity because of their increased plasma membrane exchange area, shorter transport distances within cells, and more membranous pathways for oxygen delivery to mitochondria. In support of this hypothesis, there is some evidence (though still fragmentary) indicating that small-celled *D. melanogaster* outperforms large-celled flies by maintaining flight performance when challenged by hypoxic conditions^99^ and that small-celled *Lecane inermis* is more fecund under a combination of high temperature and hypoxia than large-celled rotifers^12^. Furthermore, while *D. melanogaster*, with relatively small cells, has been shown to present higher tolerance to acute and intense heat stress, flies with larger cells tolerate chronic and mild heat stress better^100^. From this perspective, cell size orchestration throughout a body may help to coordinate the capacities of different organs so that a whole organism achieves maximal performance in a given environment with the lowest possible cost. At the same time, tissues and organs with a certain type of physiological activity can be expected to obtain different benefits from a given change in cell size compared to tissues with other types of activities^14^. This can aid in the understanding of some irregularities in systemic cell size patterns. For example, while rearing in a warmer environment was shown to result in decreased sizes of muscle and epidermal cells in the land snail *Cornu aspersum*, hepatopancreatic cell size increased^101^. Similarly, while the size of muscle cells and hepatocytes was shown to change in conjunction with body size in *Paroedura picta* geckoes, this was not the case for tracheal chondrocytes, duodenal enterocytes, renal proximal tubules, or epidermal skin cells^15^. In mice, divergent selection leading to a high basal metabolic rate led to decreases in the size of epidermal skin cells and erythrocytes, while kidney proximal tubule cells, hepatocytes, and duodenum enterocytes increased in size^55^.

In summary, we have gathered exceptionally rich information on the effects of *rictor*^*Δ2*^ and *Mnt*^*1*^ mutations on cell size in different *D. melanogaster* tissue types. These results indicate that altered activity of TOR/insulin pathways by these mutations can lead to highly correlated changes in cell size in different tissue types. We suggest that such synchrony can result in benefits from matching cell activity in different tissues and organs. At the same time, against this general tendency among different cell types, we found some autonomy in cell size determination, which occurred in the indirect flight muscles. Mechanisms of this autonomy and functional significance require further investigation. A better understanding of potential links among cell size, tissue function and the performance of an organism in the environment is crucial for advancing our understanding of fundamental ecological and evolutionary phenomena, such as mechanisms involved in structuring the geographical distribution of organisms and the sensitivity of organisms to environmental fluctuations, especially in the face of ongoing anthropogenic climate changes.

## Supplementary Information

Supplementary Information 1.

Supplementary Tables.

## Data Availability

The raw data have been uploaded in the Supplementary Materials.
