# Supplementary material for: Systemic changes in cell size throughout the body of Drosophila melanogaster associated with mutations in molecular cell cycle regulators
Source: Sci Rep. 2023 May 9;13:7565. doi: 10.1038/s41598-023-34674-y (PMC10169805; doi:10.1038/s41598-023-34674-y)
Supplement: Supplementary file 2 — Supplementary Tables. [file 41598_2023_34674_MOESM2_ESM.docx]

**Supplementary materials**

Systemic changes in cell size throughout the body of *Drosophila melanogaster* associated with mutations in molecular cell cycle regulators

Valeriya Privalova^1^, Anna Maria Labecka^1^, Ewa Szlachcic^1^, Anna Sikorska^1^, Marcin Czarnoleski^1*^

^1^Life History Evolution group, Institute of Environmental Sciences, Faculty of Biology, Jagiellonian University, Gronostajowa 7, 30-387 Kraków, Poland

VP: 0000-0002-8496-7352

AML: 0000-0002-8810-7093

ES: 0000-0002-4179-4068

AS: 0000-0003-1668-2264

MC: 0000-0003-2645-0360

*Corresponding author: Marcin Czarnoleski

E-mail: marcin.czarnoleski@uj.edu.pl

**Tables**

Table 1S. The number of flies used for the cell size measurements in legs, wings, eyes, and flight muscles per four genetic groups of *Drosophila melanogaster* (*rictor ^Δ2^*, control to *rictor ^Δ2^*, *Mnt^1^*, and control to *Mnt^1^*) males and females (where applicable).

|  | **Number of observations** | | | | | | | |
| --- | --- | --- | --- | --- | --- | --- | --- | --- |
|  | ***rictor ^Δ2^*** | | **control to *rictor ^Δ2^*** | | ***Mnt^1^*** | | **control to *Mnt^1^*** | |
|  | **male** | **female** | **male** | **female** | **male** | **female** | **male** | **female** |
| Epidermal cells in the legs | 10 | 10 | 10 | 10 | 10 | 10 | 10 | 10 |
| Epidermal cells in the wings | 10 | 10 | 10 | 10 | 10 | 10 | 10 | 10 |
| Ommatidial cells in the eyes | 10 | 10 | 10 | 10 | 10 | 10 | 10 | 10 |
| Cells in the flight muscles | 12 | N/A | 10 | N/A | 11 | N/A | 9 | N/A |

Table 2S. Descriptive statistics showing proxy measurements of cell size in legs (µm^2^), wings (µm^2^), eyes (µm^2^) and flight muscles (µm^2^) of the four groups of *Drosophila melanogaster* (*rictor ^Δ2^*, control to r*ictor ^Δ2^*, *Mnt^1^*, and control to *Mnt^1^*) male and female (where applicable) flies.

|  | **Pooled data** | | | | **Mean values per group** | | | | | | | |
| --- | --- | --- | --- | --- | --- | --- | --- | --- | --- | --- | --- | --- |
|  |  | | | | *rictor ^Δ2^* | | control to  *rictor ^Δ2^* | | *Mnt^1^* | | control to  *Mnt^1^* | |
|  | mean | sd | min | max | male | female | male | female | male | female | male | female |
| Epidermal cells in the legs | 413.13 | 72.64 | 276.7 | 718.6 | 340.83 | 395.7 | 408.11 | 444.03 | 447.36 | 503.83 | 373.3 | 391.78 |
| Epidermal cells in the wings | 166.87 | 20.17 | 128.64 | 217.08 | 136.2 | 148.1 | 158.26 | 183.63 | 169.15 | 197.1 | 161.58 | 181 |
| Ommatidial cells in the eyes | 243.69 | 26.88 | 190.9 | 291.96 | 200.65 | 211.26 | 237.72 | 245.26 | 264.18 | 282.15 | 247 | 261.3 |
| Cells in the flight muscles | 311.92 | 71.02 | 175.94 | 500.94 | 271.99 | N/A | 308.72 | N/A | 317.79 | N/A | 361.54 | N/A |

Table 3S. (a) Results of the Kruskal‒Wallis test for the size of flight muscle cells in four genetic groups of *Drosophila melanogaster*: *rictor ^Δ2^*, control to *rictor ^Δ2^*, *Mnt^1^*, and control to *Mnt^1^*. (b) Results of the Wilcoxon test for pairwise comparisons.

| **a** | **H** | 10.16 | **b** |  | ***p*** |
| --- | --- | --- | --- | --- | --- |
|  | **df** | 3 |  | control to *rictor ^Δ2^* vs. *rictor ^Δ2^* | 0.557 |
|  | ***p*** | 0.017 |  | *Mnt^1^* vs. *rictor ^Δ2^* | 0.013 |
|  |  |  |  | control to *Mnt^1^* vs. *rictor ^Δ2^* | 0.011 |
|  |  |  |  | control to *rictor ^Δ2^* vs. *Mnt^1^* | 0.557 |
|  |  |  |  | control to *Mnt^1^* vs. control to *rictor ^Δ2^* | 0.422 |
|  |  |  |  | control to *Mnt^1^* vs. *Mnt^1^* | 0.555 |
